# Humanism in clinical education: a mixed methods study on the experiences of clinical instructors in Iran

**DOI:** 10.1186/s13010-020-00088-1

**Published:** 2020-07-28

**Authors:** Hakimeh Hazrati, Shoaleh Bigdeli, Vahideh Zarea Gavgani, Seyed Kamran Soltani Arabshahi, Mozhgan Behshid, Zohreh Sohrabi

**Affiliations:** 1 Center for Educational Research in Medical Sciences (CERMS), Department of Medical Education, School of Medicine, Iran University of Medical Sciences, Tehran, Iran; 2 Medical Library and Information Science, Department of Medical Library and Information Sciences, School of Management and Medical Informatics, Tabriz University of Medical Sciences, Tabriz, Iran; 3 Nursing Education, Research Center of Medical Education, Department of Medical- Surgical Nursing, Faculty of Nursing and Midwifery, Tabriz University of Medical Sciences, Tabriz, Iran

**Keywords:** Humanism, Clinical professor, Scientometrics, Qualitative study

## Abstract

**Background:**

Medical education is currently more considerate about the human dimension. The present qualitative study aimed to explain the experiences of clinical professors with regard to humanism in clinical education in Iran.

**Methods:**

This mixed methods study had two phases, a quanitative phase of scientometrics and a qualitative phase of a content analysis. In the scientometrics phase, Ravar PreMap and VOSviewer software programs were utilized for plotting the conceptual networks. The networks were analyzed at the micro-level based on centrality indices (closeness, degree, and betweenness). The conceptual network was plotted and the prominent topics in clinical education were identified using co-word analysis. In the second qualitative phase on the topic, based on the scientometrics phase, semi-structured interviews were conducted with clinical professors. The interviews were transcribed verbatim and analyzed.

**Results:**

On the basis of the analysis of titles, abstracts, and keywords of the retrieved articles on clinical education from ISI Web of Science, Scopus, and PubMed, 1412 keywords were extracted. After the refining process, 356 keywords with 6741 relations remained. Upon plotting the conceptual network, 19 conceptual clusters related to clinical education were obtained. Then, micro-level network analysis (centrality criteria) indicated that the keyword *humanism* with the frequency of 137 had the highest rate (97.753), closeness (97.802), and betweenness (13.407). Moreover, from the interview data analysis, two themes of “intertwined nature of the human spirit in clinical education” and “humanistic behavior of professors in clinical education” were extracted.

**Conclusion:**

As a part of the educational culture, humanistic values must be intertwined with the medical education curriculum. In this regard, humanism and clinical reasoning are the two major clusters of clinical teaching; moreover, altruism and adherence to humanistic values, and scientific qualification are other main pillars that should be considered as the criteria for the selection of clinical professors and medical students.

## Background

Why is humanism in medicine gaining momentum once more in the present era? Is it an appropriate time to regard humanism as a major concern of medical education? In the past four decades, the spread of new technologies in medical sciences for diagnosis has made doctors dependent on laboratory research and technology, while psychological status of the patients, and moral and social aspects were ignored in their diagnosis, treatment, and physical well-being [1]. In addition, the required increase in productivity, hectic work schedules and imposed diverse responsibilities, competitive work settings, governmental pressure, and administrative bureaucracy in healthcare have limited commitment of physicians to the humanistic dimension of medicine, thus disrupting the humanistic spirit [2]. Mere scientific attention to medical functions, and ignoring humanistic and moral aspects in doctor-patient relationship, has seriously damaged professionalism and the quality of services. However, to render services in medicine [3]; one must deal with patients who need care [4] and ask for attention of doctors and their rapport to assist the patients by explaining their physical, psychological, and emotional states and concerns [5]. Therefore, a good doctor should possess not only medical knowledge and skills, but also sound ethical judgment and behavior, and compassion in order to secure the trust of patients and their families [6, 7]. While establishing relationships with patients, doctors must consider the fact that the patients have a body, a soul, feelings, and expectations [8], and the disease belongs to a living human being with specific lived experiences of the diseases which play a critical role in diagnosis and acceptance of treatment. In the words of Sir William Osler, knowing *who* has the disease is more important than knowing *what type* of disease he has; “Listen to the patient; he will tell you what the diagnosis is” [9].

In this regard, professionalism in medicine, includes customs, beliefs, communication styles, functions, and thought processes that are transferred from professors to students through the hidden curriculum (unofficial norms) [10], professor role modelling, and student observation of social conducts and habits of the professors and staff in the clinical settings. Medical students observe the behavior and performance of their clinical professors and thus form their professional identity [10]. To allow patients to express their emotions, the clinical professor, listens to, establishes eye contact, and touches patients; more, adopts an appropriate tone and pace of speech [11] and shows empathy with them [12, 13], thereby explicitly teach students how to establish good rapport and appropriate personal communication with patients. Clinical professors with a humanistic view and empathy can act as positive role models. On the other hand, the negative role model set by professors, harms the students’ emotions, attitudes, and behavior [2]. Thus, the role and experiences of professors with regard to humanistic behavior in clinical education are of special significance; however, it seems that medical education currently ignores paying attention to these significant dimensions of medicine studies To the best of our knowledge, the literature review yielded no study examining humanism in clinical education from the viewpoint of clinical professors in Iran. Therefore, this mixed method study of scientometrics and a qualitative content analysis phases was attempted to explore the experiences of clinical professors with regard to humanistic behavior such as honesty, integrity, caring, compassion, altruism, empathy, and respect for self, patients, peers, and other health care professionals in clinical education in Iran as a unique context wherein education and treatment are intertwined.

## Methods

In the scientometrics phase, data mining was employed to determine the conceptual clusters in medical education. The studied population comprised all published scientific articles on clinical education indexed in the ISI Web of Science, Scopus, Medline/PubMed, and Medline databases from January 1980 to October 2018.We limited the beginning of the search to this era since in the 1980, the second wave of reforms in universal medical education was performed on the basis of the Flexner’s report. The reforms led scientists to comprehend the difference between andragogy and pedagogy learning and concepts such as student-centeredness, integration in medical education, and problem based learning, and community-oriented medical education entered medical education [14] .

In this regard, articles were searched and retrieved using the following keywords combined with Boolean operators:

(((“Hospitals, Teaching”[Mesh] OR “Teaching Rounds”[Mesh] OR “Clinical Education”[Text word] OR “Morning Report”[Text word]) AND “Medical Students”[Text word]) AND “1980/01/01”[PDAT]: “2018/04/31”[PDAT]) AND English [Lang]))

Searches were limited to the fields of titles, keywords, and abstracts. Ravar PreMap (a free and native software for identifying co-occurrence of words and preparing data for Mapping, designed by Dr. Mohammad Tavakolizadeh, 2007 was used (http://mravari.blogfa.com), and VOSviewer software program, v.1.6.11, free and easy to use software for visualizing bibliometric network (https://www.vosviewer.com), were used for plotting the conceptual networks. The networks were analyzed at the micro-level based on centrality criteria (closeness, degree, and betweenness) [15, 16]. Closeness demonstrates the mean length of the shortest path from among the paths between the nodes of a network. Nodes with a high closeness index have larger effects on the network and play a central role in it [15]. Degree demonstrates the mean length of the shortest path among the paths between the nodes of a network. Nodes with a high closeness index have stronger effects on the network and play a central role in it [16] . Nodes with a high betweenness criterion play an important role in network connections and have a central role in the network [15, 16].

The data retrieved from the databases were transferred to Excel in order to determine the overlapping articles and to delete duplicates. Then, to determine co-occurrence of the words, as well as merging and standardizing of the keywords, they were entered into Ravar PreMap to identify keywords with a high frequency to filter the words, and thus to select the most relevant ones. This was done in five steps: 1) merging singulars and plurals of the words, 2) re-writing abbreviations in the complete form, 3) eliminating keywords with a general meaning and no semantic load standardized synonyms, and 4) converting specific keywords into general keywords, and 5) deleted numbers or keywords such as %, #, &, >. The MeSH list was used to select general keywords and the most frequently used synonyms of the words. Words were separately standardized by two researchers and finalized upon consensus. The co-word analysis method was adopted in order to plot the conceptual network and identify the prominent topics in clinical education, which is a content analysis method aiming to identify the map of science and major topics in different domains of science [17]. To this end, automatic text mining methods were adopted, whereby the output of Ravar PreMap was inputted into VOSviewer and conceptual clusters were plotted.

The second phase of the study involved qualitative conventional content analysis. In this phase, purposive sampling, continued by theoretical sampling were used. The data were collected from the key participants with rich experiences about the topic. Semi-structured interviews were conducted with selected clinical professors some of which had Education Development Center (EDC) management experience in Shahid Beheshti, Tabriz, Isfahan, Shiraz, Mashhad, Mazandaran, and Guilan Universities of Medical Sciences. A list of guided questions was designed to cover all relevant topics. However, the interview questions were not fixed, and the interviews were guided and probed according to the issues emerged from the interaction between the interviewer and the interviewees. More, the relevance of questions to the objectives of the study was taken into account. Before starting the interviews, an information sheet was presented to the study participants, and the study objectives, voluntary participation, and confidentiality of the data were emphasized. Afterwards, their informed consent was secured, and the interviews were conducted and recorded. After the interview sessions, each interview was transcribed verbatim and analyzed to identify key humanistic attitudes of clinical educators. Then, the data were carefully read and re-read for several times and the first-level coding with an emphasis on the manifest and implied content was extracted by identifying and highlighting the units of analysis, namely sentences and paragraphs. A code was allocated to each unit of analysis. Subsequently, the codes were classified based on their scope and attributes into sub-categories and categories. The codes were repeatedly checked and contradictions were resolved by discussion. To determine the validity of the data, repeated examination of data and simultaneous analysis were performed. The extracted codes were examined by the participants and their acceptability was ensured. The research team reached a consensus on the extracted codes and thus confirmed the process of data analysis. Moreover, immersion was performed upon continuous long-term involvement with the data. To ensure transferability, the details of the study were carefully documented and the steps were explained in details in order to allow the external audit.

## Results

The search strategy identified 3939 articles. After removal of duplicates in the databases and the assessment of title and abstracts, 1229 articles were included into the scientometrics analysis.

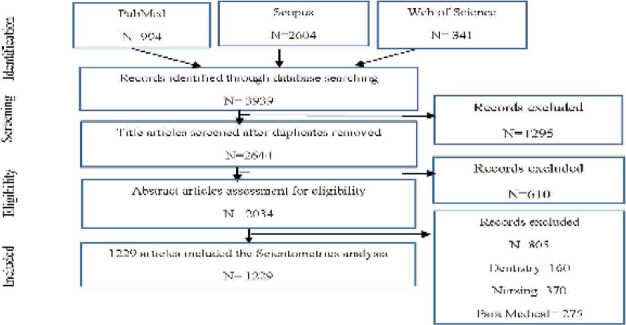



PRISMA flowchart of articles selection

**Fig. 1:**
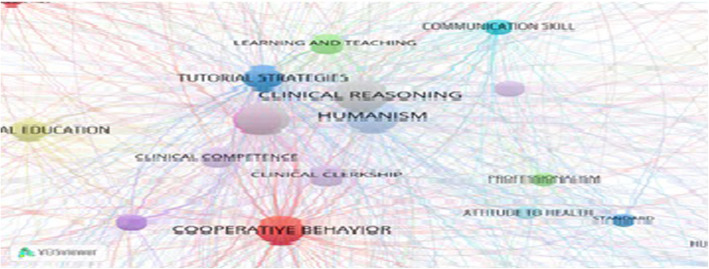
Co-occurrence network of keywords in clinical teaching research

1412 keywords resulted from the analysis of the titles, abstracts, and keywords of the articles on clinical education retrieved from PubMed, Scopus, Web of Science databases, using the Ravar PreMed. After the refinement process, 356 keywords with 6741 relations remained. The keywords with minimum frequency (1 and 2) were discarded. Next, using VOSviewer, content analysis was performed, yielding 19 conceptual clusters in the domain of clinical education (Fig. 1).

Following the micro-level network analysis (centrality criteria), the keyword humanism with the frequency of 137 had the highest degree (97.753), closeness (97.802), and betweenness (13.407). Ten keywords with maximum frequency, betweenness, and degree are presented in Table 1.

**Table 1:** Ten keywords with maximum frequency, degree, and betweenness in clinical education

N	Keywords	Frequency	Betweenness	Degree	Closeness
1	Humanism	137	13.407	97.753	97.802
2	Clinical reasoning	139	13.213	95.506	95.699
3	Clinical education	129	11.208	92.697	93.194
4	Cooperative behavior	102	7.980	83.708	85.990
5	Medical education student	105	7.716	82.865	85.372
6	Tutorial strategies	72	4.411	69.101	76.395
7	Medical education	59	3.287	61.236	72.065
8	Clinical clerkship	62	3.125	60.674	71.774
9	Learning and teaching	50	2.174	52.247	67.681
10	Clinical competence	49	2.145	54.213	68.593

### Results of the qualitative phase

Semi-structured interviews were conducted with 27 clinical professors (six were EDC managers at Iran, Tabriz, Mazandaran, Shahid Beheshti, Mashhad, and Shiraz Universities of Medical Sciences; five were professors at the department of surgery; four at the department of psychiatry; three at the department of emergency medicine, and 12 at the department of internal medicine, including gastroenterology, rheumatology, endocrinology, and ENT). Four participants were full professors, while 26 were associate professors. Based on the analysis of the interviews, two themes of “intertwined nature of the human spirit in clinical education” and “humanistic behavior of professors in clinical education” were extracted.

In the theme “intertwined nature of the human spirit in clinical education”, two categories of “humanistic values in clinical education” and “patient as the axis for clinical education” were extracted (Table 2). For the category “humanistic values in clinical education”, Participant 3 stated, “Humanity is intertwined with medicine; one cannot treat a patient while ignoring his/her human dimensions. The patient trusts us.” Participant 7 mentioned: “We deal with human life. The patient has rights he/she may not be aware of. The professor is a role model for the student. When students observe that their professors talk with patients kindly and behave with patience, they view humanitarianism as their responsibility. I had a patient who was hopeless, and whose mother did not trust the healthcare team. He felt that he was dealt with as an education scenario, and did not let any student to examine him. I talked with him and his mother every day, half an hour before visiting him, and explained the steps of treatment so that they would collaborate with the students. I think the students learned a lot by observing how I treated them; they learned that respectful behavior and gaining trust of the patient are significant in the process of treatment.”

**Table 2:** Categories and sub-categories for the theme “intertwined nature of the human spirit in clinical education”

Open code	Category	Theme
Dealing with the health of society Importance of human relationship in clinical education for patients Acquisition of humanitarian and humanistic skills through clinical education Intangibility of treatment errors for all and importance of attention to patient rights	Humanistic values in clinical education	**Intertwined nature of the human spirit in clinical education**
Patients has the central role in problem identification and hypotheses generation and initiator of education Patient as the source of original information Patient as the educational content Patient-centeredness in clinical education instead of disease- and reference-centeredness Uniqueness of each patient and the need for diagnoses tailored to the patient needs Respecting autonomy and rights of patients while educating students	Patient as the axis of clinical education	

In the category of “patient as the axis of clinical education”, Participant 1 remarked, “Patients are noble. If they do not cooperate with us, we could not teach our students anything.” Participant 13 said: “The patient is our educational content. Each patient is unique, with unique characteristics. Two people may have the same disease, but the same treatment cannot be selected for both of them. The student must observe that it is the *patient* who matters, not the disease.”

In the theme “Paying attention to human dignity of patients”, “Respecting patient rights”, “Creating a sense of empathy with patients”, “Not making patients anxious”, and “inculcating a sense of priority of treatment over education” were identified. (Table 3). In the category “attention to and respecting the human dignity of patients’”, Participant 25 mentioned: “The students first mistake is that they do not introduce themselves, and the patient realizes that these are still students, so they sometimes give false information or verify the students’ knowledge.” According to Participant 19, “If you behave amiably with patients and greet them, you can gain their trust and ensure their cooperation.” Participant 20 said, “I always try to memorize my patients’ names, especially those of the elderly or children. When you call them by their first name, they feel intimate with you and you can establish a good rapport with them.” In the category “respecting patients’ rights”, 13 recounted: “When I visit a patient in the ward for the first time, I introduce myself and tell him/her I am the doctor in charge and these are my students. You can tell them any problem you have and they will inform me if anything goes wrong.” According to Participant 7, “Some diseases are related to the patient’s genitals. We must be careful about these cases.

**Table 3:** Categories and sub-categories of the humanistic behavior of clinical professors

Open code	Category	Theme
Behaving respectfully with patients Respecting patients’ boundaries and using media for explaining some of the subjects and to avoid repeated examinations by the students. Respecting and gaining the trust of patients Assisting patients when needed for winning their trust Introducing students as part of the healthcare team and the link between patients and doctors Introducing students to patients as facilitators of the treatment process Introducing students and explaining the educational nature of the round Not regarding patients as educational and research content Holding short rounds in order not to tire the patients out Appreciating patients for accepting the role of an educational case Paying attention to patients’ human dignity in grand rounds Behaving humanely with patients Behaving amicably with patients Viewing the patients as having the same status as the doctor and providing convincing explanations a Paying attention to humanism and respecting human dignity in dealing with patients and education Introducing students and asking the patients to cooperate with them Behaving affably with patients and following-up for their main complaint	Paying attention to human dignity of patients	**humanistic behavior of professors in clinical education**
Seeking the permission of patients for performing physical examination by students Explaining the educational nature of the clinic to patients Asking for patients’ consent and behaving respectfully with them Examination by few students in order to respect the patients’ rights and not giving them the wrong idea Asking patients’ permission for any medical intervention Paying attention to patient autonomy and rights in the educational session Visiting patients when they are alone, and respecting their privacy Keeping patients’ secrets Respecting patients’ individual rights and asking for their permission for taking pictures with them. Professors performing the examination and history taking if the patients do not consent to being examined by students Introducing oneself, explaining the educational nature of the visit, and asking the patients’ permission Holding discussions inside each cabin for respecting the confidentiality of patient information Explaining treatment priorities for critically ill patients after performing medical interventions Holding discussions inside each patient’s cabin for respecting their privacy Holding discussions in the presence of patients only if their health allows it and they consent to it Holding discussions with students outside the ward in case of patients’ lack of consent or poor psychological condition Confidentiality, respecting privacy, self-control, and management of self-conflicts Involving patients in decision-making on the types of treatment and medical interventions Directing students’ attention to patients’ family and conditions when teaching	Respecting patient rights	
Not leaving patients alone if a problem arises Accepting one’s mistakes in case of complications arising for patients Accepting patients with complications Creating a sense of compassion and providing as many services to patients as possible Establishing primary communication with patients and gaining their trust Establishing a friendly relationship with hopeless and sad patients Helping patients if their entourage is not available Calling patients by their name and greeting them in their ethnic language for securing their trust	Creating a sense of empathy with patients	
Not holding long discussions with clinical terms in the presence of patients in order not to make them anxious Teaching students while taking the patient’s presence into consideration Regarding patients and their concerns important during educational rounds Paying attention to the patient’s stress during educational rounds Giving information to patients on the steps of treatment and the measures taken Holding discussions outside the patient’s room in order to respect their anxiety and concerns Speaking to patients in their own language when explaining their disease to them Raising patients’ awareness of invasive measures in order to reduce their anxiety Using academic terminology for preventing anxiety and stress in patients Informing patients of their health status and length of hospital stay based on the preliminary diagnosis Introducing the doctor in charge and ensuring the patients that the doctor has undertaken the responsibility for their treatment Ensuring patients of their status and recovery Explaining the status of the disease to patients or their entourage Explaining the reason for examination by another specialist in order to reduce patients’ anxiety	Not making patients anxious	
Follow-up in order to expedite patients’ treatment Prioritizing treatment over education due to ethical considerations Prioritizing treatment over education in the case of emergency and critically ill patients Ensuring patients of the follow-up for their problems Inculcating the priority of treatment over education in students Ensuring patients by repeated examinations in order to help their treatment Making the patients feel the priority of treatment Making the patients feel that they benefit from clinical education Establishing relationships with patients and ensuring the follow-up for their issues	Inculcating the priority of treatment over education	

If, for example, the patient is a young girl, she may feel uncomfortable, and so we must not allow students to examine her.” According to Participant 3, “Sometimes patients prefer to consult us directly. In our field, we have patients with psychiatric diseases who may not cooperate with us. In these cases, we must play the role of consultant by ourselves. Participant 15 said: “Several patients may be in the same room. When we examine one of them, we must draw the curtain so that he/she would not be uncomfortable. The patient may not want other patients to hear him/her. We must be careful with these issues.” In the category “creating a sense of empathy with the patients”, Participant 5 said: “You see sadness and loneliness in a patient’s face. If you greet them and ask about their health and tell them that you are there for them and there is no need to worry, you can give them hope. The students observe your behavior.” Participant 17 remarked: “When doing the rounds, the patients’ next of kin is usually asked to leave the ward. Some patients cannot take care of themselves. I tell my students, ‘If you see that patients need help, help them. Take their hands. If they want to take their medicine, give them a glass of water’. These actions gain their trust and ensure their cooperation.” According to Participant 12, “Sometimes a patient has to be hospitalized an extra day for getting the results of a test. I sign the discharge form and tell them to ask someone else to get the results and take them to me the next day. Patients have families who are worried about them. Therefore, I speed up their discharge process so that they can go back to their families.”

In the category “inculcating a sense of priority of treatment over education”, Participant 15 mentioned: “I always tell my students that the patients are always right, even if they get mad or don’t let you examine them. Respect them; they are under no obligation to be a scenario for your education. Their treatment comes first.” According to Participant 16, “Some people think that we can use patients as a scenario for education in any way they desire and that students are allowed to examine them as many times as they want. However, patients are under no such obligation. The students can examine them only if they allow them to.” Participant 9 said: “Some patients feel that teaching hospitals view them as clinical scenarios, and their treatment does not come first. I tell my students to follow up the patients and ensure them of this follow-up. Occasionally I call the imaging center when the student is present and follow up the patient CT scan. The students realize that the doctor is responsible for the patient follow-up, and the patient peace assures that his/her treatment is moving forward.” As for the theme “taking care not to worry the patients”, Participant 10 said: “The Internet has recently become another stressor for patients. When we discuss the disease in the presence of the patients, they check the new terminology they hear, which may not even be related to their disease. I always try to postpone extra discussions with students to the end of the rounds at the corridor or in the class.”

## Discussion

The present study aimed to explore the experiences of clinical professors with regard to the critical topic of humanism and humanistic behavior in clinical education settings, for which two themes of “intertwined nature of the human spirit in clinical education” and “humanistic behavior of clinical professors” were extracted.

In the theme “intertwined nature of the human spirit in clinical education”, two categories of “humanistic values in clinical education” and “patient as the axis for clinical education” were extracted. In the majority of studies, medicine has been regarded as a science and an art and it has been explained that professionalism cannot be attained through mere medical knowledge [18]. Today, most clinical professors depend on para-clinical (Radiology-CT Scan, Laboratory, Pathology) results and medical equipment for making a diagnosis. Occasionally para-clinical errors lead to medical errors [19]. The art of medicine combines the data coming from medical examination, patient history, and para- clinical results in order to make the most probable diagnosis [18]. In the words of Sir William Osler, clinical education without patient is like sailing without going to sea, because each patient is a unique human being with specific qualities which affect the process of diagnosis and treatment. Some doctors may regard patients as clients and ignore their humane aspects [13, 20]. This view of the medical profession as an absolute science and ignoring the humane dimension in clinical encounters lead to the gradual deterioration of doctor-patient relationship [3]. Humanistic behaviors help doctors solve communication problems, transfer information to patients, understand their views, and respect their rights [21]. Studies show that doctors with humanistic behaviors are more successful in encouraging patient cooperation in the process of treatment [22]. Students with an optimal degree of humanistic behavior perform better in history taking and physical examination compared to other students [23].

Deliberate or inadvertent denial of intrinsic values of patients, threatens their human dignity, and leads to their non-cooperation, ineffective communication, which deteriorates humanistic encounters with patients. According to Spiro, an “unseen and unheard patient” shows that we face the problem of “de-humanization” and must re-humanize medicine [24].

In the theme “humanistic behavior of professors in clinical education”, five categories of “Paying attention to human dignity of patients”, “Respecting patient rights”, “Creating a sense of empathy with patients”, “Not making patients anxious”, and “inculcating a sense of priority of treatment over education” were extracted.

Respecting human dignity of the patients can be summarized in the saying “Do unto others as you would have them do unto you” [25]. Paying attention to psychological needs of patients such as keeping their peace of mind, empathizing with them, understanding their emotions, preferences, interests, needs, wants and desires, supporting them and establishing rapport based on mutual trust, and creating respectful and intimate human relationships are the requirements of rendering professional care to the patients [25]. A doctor who respects beliefs and attitudes of his/her patients, involves them in clinical decision-making, attends to their psychological states, regards them as a unique beings, considers their familial, social, and physical contexts, possesses proper communication and listening skills, creates a sense of trust in them, and behaves with warmth and empathy has regarded humanistic behaviors as part of his/her professional performance values [23].

The quality of clinical care is influenced by humanitarianism and job satisfaction. Research has shown that doctors with a higher degree of empathy with patients have had higher job satisfaction and fewer clinical errors which lead to lawsuits [26]. Moreover, when patients receive empathy from doctors, their positive clinical outcomes increase [23]. This increased trust and mutual understanding encourages patients in further cooperation, expressing their symptoms, and giving more precise information, thus contributing to a successful treatment [23, 27]. Professors who empathize with their patients and are happy when visiting them, enjoy their job as skillful individuals who will not suffer from occupational burnout [28].

Another category was respecting rights of patients in clinical education. Patients demand respect for their privacy, values, and expectations and do not wish to be viewed as educational scenarios or subjects of study. Unfortunately, in most studies on patient rights, patients were dissatisfied with repeated examinations, the doctors and the healthcare team who do not introduce themselves, and lack of respect for their privacy [29], for example failing to draw the curtain during examination, failing to ask their permission or appreciate their cooperation in the teaching process [30, 31]. Keeping patients’ secrets and respecting their privacy are manifestations of respecting their human dignity [25, 32]. Handerson et al. enumerated other issues such as failing to cover the patients’ body while taking them to the bathroom, meddling with their private life, and asking unnecessary questions as examples of disrespecting the patients’ humane value [33].

In Iranian teaching hospitals, the healthcare team believes that patients who consented to receive treatment from students or residents are educational scenarios, while this is against the charter of patients’ rights; the educational encounter with patients requires their permission [32] and understanding that they have no obligation to be treated as subject of studies [34].

Humanitarian values in clinical settings are mostly transferred to students through role modeling position of professors and the hidden curriculum [10, 35–37]. It is expected that human virtues, including humanitarianism, compassion, and empathy, be explicitly enhanced while offering medical care [38]. Sometimes, however, these values are contradicted in medical cultures governing the medical practice [36]. When a clinical professor does not respect humanistic values, students face contradictions, tend to view patients objectively and technically, and ignore humanism in dealing with them [6, 39, 40]. In the study by Billingess et al., it has been mentioned that students in the clinical settings observe numerous unprofessional behaviors as well as lack of empathy and humanistic behaviors with patients and assume that humanistic behaviors are not part of their professional role [41]. The professor’s role is of utmost importance in inculcating students with humanism in clinical education. Thus, the academia must plan for the selection of clinical professors and invest in the enhancement of humanistic behaviors in clinical education so that humanism would restore its status in medicine by empowering the professors [37].

## Conclusion

The curriculum of medicine must highlight humanistic values which form the student professional identity, and respectful encounter with the patients must be recognized as a norm in medicine. It is essential to commit doctors and medical students on spending ample time with patients, listening to them carefully, being sensitive to their emotional states, and regarding humanistic beliefs as the spirit governing their professional activity. Medical boards have an obligation to regard humanistic elements as an important component of clinical competency, because physicians should incorporate attitudes that value the patient as a human being.

### Limitations and resolving them

We acknowledge that the small sample in this qualitative study may not be plentifully representative of the clinical teachers in undergraduate medical education in Iran. However, to increase the diversity of participants, medical faculties and EDC managers from across the country were interviewed to reach maximum variation and to resolve the inconsistencies.

## Data Availability

Data are available from the authors upon reasonable request and with agreement from Iran University of Medical Sciences Vice-Chancellor for Research.

## Appendix

**Table 4:** PubMed/Medline search results

Search	Query	Items found	Time
#1	Search ((“Hospitals, Teaching”[Mesh] OR “Teaching Rounds”[Mesh] OR “Clinical Education”[Text word] OR “Morning Report”[Text word]))	54305	09:59:02
#2	Search “Medical Students”[Text word]	50198	09:58:03
#3	Search (#1 AND #2)	1315	09:56:31
#4	Search (((“Hospitals, Teaching”[Mesh] OR “Teaching Rounds”[Mesh] OR “Clinical Education”[Text word] OR “Morning Report”[Text word]) AND “Medical Students”[Text word]) AND “1980/01/01”[PDAT]: “2018/04/31”[PDAT]) AND English [Lang]))	994	09:53:46

## Abbreviations

EDC: Educational Development Center
ENT: Ear,Nose, Throat
Ravar PreMap software: This native software has been designed to identify Co-occurrence Word and preparing data for Mapping by Dr. Mohammad Tavakolizadeh, 2007.
VOSviewer software: designed to construct and visualize bibliometric maps such as co-authorship, co-occurrence, and citation based maps by Van Eck and Waltman, 2009.
